# Supplementary material for: A hybrid framework with large language models for rare disease phenotyping
Source: BMC Med Inform Decis Mak. 2024 Oct 8;24:289. doi: 10.1186/s12911-024-02698-7 (PMC11460004; doi:10.1186/s12911-024-02698-7)
Supplement: Supplementary file 1 — Supplementary Material 1. [file 12911_2024_2698_MOESM1_ESM.zip › Additional file 1.docx]

**Appendix A Prompt Templates**

| Zero-Shot | Given a text, the start and end indices of a mention from the text, and the mention itself, determine whether the mention is a true rare disease mention or not. Return "yes" if it is a true mention, "no" if not a true mention. Please only answer yes or no in your response.  ### Report: {report}. Mention: {mention}. Start: {start_index}. End: {end_index}.  ### Output: |
| --- | --- |
| Few-Shot | Given a text, the start and end indices of a mention from the text, and the mention itself, determine whether the mention is a true rare disease mention or not. Return "yes" if it is a true mention, "no" if not a true mention. Please only answer yes or no in your response.  ### Report: {report}. Mention: {mention}. Start: {start_index}. End: {end_index}.  Here are some examples:  Example 1. Report: {report}. Mention: {mention}. Start: {start_index}. End: {end_index}. Answer: {groudtruth}  Example 2. Report: {report}. Mention: {mention}. Start: {start_index}. End: {end_index}. Answer: {groudtruth}  …  ### Output: |
| KAG | Given a text, the start and end indices of a mention from the text, and the mention itself, determine whether the mention is a true rare disease mention or not. Return "yes" if it is a true mention, "no" if not a true mention. Please only answer yes or no in your response.  ### Report: {report}. Mention: {mention}. Start: {start_index}. End: {end_index}.  Definition of { mention }: {definition}. ### Output: |

Table A1. Example of prompt using LLMs for rare disease identification.
